# Gapless near Telomer-to-Telomer Assembly of *Neurospora intermedia*, *Aspergillus oryzae*, and *Trichoderma asperellum* from Nanopore Simplex Reads

**DOI:** 10.3390/jof11100701

**Published:** 2025-09-27

**Authors:** Mikael Terp, Mark Nyitrai, Christian Enrico Rusbjerg-Weberskov, Teis E. Sondergaard, Mette Lübeck

**Affiliations:** Department of Chemistry and Bioscience, Aalborg University, 9220 Aalborg, Denmark; mter@bio.aau.dk (M.T.); cerw@bio.aau.dk (C.E.R.-W.); tes@bio.aau.dk (T.E.S.)

**Keywords:** telomere-to-telomere, nanopore sequencing, long read sequencing, high molecular weight DNA, fungal genomes, Snakemake

## Abstract

Assembling high-quality fungal genomes, specifically telomere-to-telomere (T2T) gapless assemblies, often necessitates the integration of multiple sequencing platforms. This requirement poses a limitation on the number of fungal genomes that can feasibly be generated within a single project. Here, we demonstrate that haplotype-aware error correction (HERRO) of Oxford Nanopore simplex reads enables the generation of high-quality assemblies from a single sequencing platform. We present an automated Snakemake workflow that, without manual intervention, produced gapless genome assemblies for industrially relevant strains: *Neurospora intermedia* NRRL 2884, *Trichoderma asperellum* TA1, and *Aspergillus oryzae* CBS 466.91, each achieving complete BUSCO (Benchmarking Universal Single-Copy Orthologs) scores exceeding 98%. Among these, only the *T. asperellum* assembly yielded a fully telomere-to-telomere gapless genome, while the *N. intermedia* and *A. oryzae* assemblies were gapless but near-telomere-to-telomere. Manual curation was required for the mitochondrial genome assembly of *N. intermedia*.

## 1. Introduction

The first whole-genome sequence of a filamentous fungus was reported in 2003 [1], a milestone that required substantial resources. Advances in genome-sequencing technologies now allow for such projects to be completed routinely within weeks, depending on the volume and type of data required. High-throughput short-read sequencing with Illumina can be performed rapidly, whereas projects requiring high molecular weight (HMW) DNA for long-read sequencing generally take longer. Nevertheless, both approaches are considerably faster and more cost-effective than the methods available in 2003 [2]. The reduction in sequencing time and cost is largely attributable to the capacity of second- and third-generation sequencing platforms to generate gigabases of data per run [3,4]. With the advent of third-generation sequencing, it has become increasingly feasible to assemble genomes to telomere-to-telomere (T2T) completeness [5]. Achieving such assemblies, however, often necessitates combining multiple complementary sequencing platforms, for example, long-read data from PacBio and/or Oxford Nanopore for assembly, short-read Illumina data for polishing, and long-range data such as Hi-C for scaffolding [6,7,8]. For Nanopore sequencing, polishing with high-quality short reads has historically been standard practice due to its higher error rate compared to PacBio [9]. The accuracy of Nanopore reads can be improved substantially, from an average Phred quality score of Q20 to Q30, through duplex sequencing, enabling the resolution of complex genomes into high-quality assemblies [10]. Accuracy can also be enhanced through pre-processing methods, such as haplotype-aware error correction (HERRO), which has recently been applied to produce T2T human genome assemblies from Nanopore simplex data alone [11], as well as four near-T2T (meaning most telomeres are assembled) assemblies of *Colletotrichum lini* strains, a pathogenic fungus of flax [12]. T2T assemblies allow for the resolution of long repetitive regions, such as telomeres and centromeres [13], and can resolve into fully gap-free assemblies in which no contigs are joined post-assembly (i.e., no ambiguous “N” gaps) [5,14]. The absence of scaffolding gaps improves gene prediction by preventing artificial fragmentation of coding sequences (CDS). This is particularly important for pathogenicity and toxicity studies, as biosynthetic gene clusters (BGCs) can span up to 220 Kb [15,16] and may be missed or fragmented in assemblies of low contiguity [17]. Because BGCs are predominantly located in subtelomeric regions [17], which were difficult to resolve with older sequencing and assembly approaches, their identification benefits greatly from T2T assemblies. Resolving BGCs, including polyketide synthases (PKSs), non-ribosomal peptide synthetases (NRPSs), terpene synthases, and ribosomally synthesized post-translationally modified peptides (RiPPs), is critical for novel drug discovery [18,19], a process now accelerated by the unprecedented pace of fungal genome sequencing. Similarly, the identification of carbohydrate-active enzymes (CAZymes) is advancing rapidly, supporting research into lignocellulosic biomass degradation [20]. Species of *Neurospora*, *Trichoderma*, and *Aspergillus* are among the most widely studied filamentous fungi [21]. In industrial contexts, *Trichoderma* and *Aspergillus* dominate patents for the production of organic acids (notably citric acid) and proteins [21], while *N. intermedia* and *N. sitophila* are recognized for their role in producing red oncom, a traditional Indonesian fermented food [22]. High-quality fungal genomes are essential for species identification [23], understanding fungal plant virulence [24], enabling drug discovery [18], and supporting the development of industrial production strains [25].

In this study, we present (i) the first near-T2T gapless genome of *Neurospora intermedia* NRRL 2884; (ii) a near-T2T gapless genome of *Aspergillus oryzae* CBS 466.91; (iii) a fully gapless T2T genome of *Trichoderma asperellum* TA1; and (iv) a Snakemake workflow for generating fungal gapless near-T2T genomes exclusively from Oxford Nanopore simplex data.

## 2. Materials and Methods

### 2.1. DNA Extraction and Sequencing

*Neurospora sitophila* (NRRL 2884) was acquired from ATCC® (36935™) and was isolated from Oncom, Indonesia. *Aspergillus oryzae* CBS 466.91 was acquired from the WI-KNAW culture collection in the Netherlands and originally isolated in Osaka, Japan. *Trichoderma asperellum* TA1 was isolated from field soil at a farm belonging to University of Copenhagen, Denmark, and initially morphologically identified as *Trichoderma harzianum* but renamed as *T. asperellum* based on ITS sequencing (unpublished results). All isolates were stored at −80 °C either as agar plugs or conidia in 15% glycerol. The acquired strain of *Neurospora sitophila* will be denominated as *Neurospora intermedia* NRRL 2884, as it was incorrectly identified when acquired from the strain collection in 2022. Revisiting the collection in 2025, it is stated that “The DNA sequences indicate that this strain is phylogenetically closer to *N. intermedia* than *N. sitophila*”, which also is confirmed in this study. The strains were grown on yeast extract peptone glucose (YPG) agar plates (Glucose 25 g/L, Peptone 20 g/L, Yeast extract 10 g/L, and Agar 20 g/L) for 5 days at 30 °C. Subsequently, 10 plugs (3 mm ø) were prepared from each plate and used for inoculation of 100 mL liquid medium in 250 mL Erlenmeyer flasks. *N. intermedia* NRRL 2884 and *A. oryzae* CBS 466.91 were grown for 2 and 5 days, respectively, in a YPG medium at 30 °C, and *T. asperellum* TA1 was grown for 2 days at 25 °C in a yeast extract sucrose (YES) medium (Sucrose 150 g/L, Yeast extract 20 g/L, MgSO_4_ 0.5 g/L, 1 mL YES trace solution (ZnSO_4_·7H_2_O 16 g/L, CuSO_4_·5H_2_O 5 g/L), pH 6.5) with agitation of 150 rpm. Mycelium was harvested by being poured into autoclaved double-layered Mira cloth (merckmillipore) and washed with autoclaved water. The harvested mycelium was frozen in liquid N_2_, then lyophilized (Scanvac Coolsafe Freeze Dryer), and ground with a mortar and pestle. HMW DNA from *N. intermedia* NRRL 2884 and *A. oryzae* CBS 466.91 was extracted with the phenol–chloroform method and purified, as described in [26]. To prepare HMW DNA, 90 mg of lyophilized ground mycelium was added to each of four 2 mL Eppendorf tubes. To each tube, 1200 μL of extraction buffer (100 mM Tris-HCl, pH 8.0, 20 mM EDTA, 0.5 M NaCl, and 1% SDS) and 700 μL of UltraPure™ Phenol:Chloroform:Isoamyl Alcohol (25:24:1, *v*/*v*) (Thermo Fisher Scientific, Waltham, MA, USA) were added. The mixture was combined by flicking the tubes until the mycelium was fully wetted, followed by 10 min of mixing on a HulaMixer (Thermo Fisher Scientific) at room temperature. Mycelium was then pelleted by centrifuging at 14,100× *g* RCF (Eppendorf MiniSpin, Hamburg, Germany) for 5 min. The aqueous phase from each of the four tubes was transferred to new 2 mL Eppendorf tubes. Subsequently, 4 μL of RNase A (100 μg/mL) (Qiagen, Valencia, CA, USA) was added to each tube. The tubes were mixed by inverting 10 times and then incubated at 50 °C for 30 min. After incubation, in a 1:1 ratio of UltraPure™ Phenol/Chloroform/Isoamyl, alcohol was added, and the samples were mixed by inverting 10 times. The samples were then centrifuged at 14,100× *g* RCF for 5 min. The aqueous layers from all four tubes were combined into a single 15 mL tube and loaded onto a QIAGEN Genomic-tip 20/G, which had been equilibrated with 1 mL of QBT from the Genomic Buffer Set (Qiagen). The Genomic-tip was washed three times with 1 mL of buffer QC. HMW DNA was then eluted with 1 mL of buffer QF. The DNA was precipitated by adding 700 μL of isopropanol and incubating at room temperature for 30 min. After incubation, the sample was centrifuged at 14,100× *g* RCF for 15 min. The supernatant was discarded, and the pellet was washed twice with 1 mL of ice-cold ethanol, with centrifugation at 14,100× *g* RCF for 5 min after each wash. After the final ethanol wash, the supernatant was discarded, and the pellet was air-dried at room temperature. The DNA pellet was dissolved in 65 μL 10mM Tris-HCl buffer pH 8.5, and the sample was left on the HulaMixer for 1–7 days to ensure complete dissolution. HMW DNA extraction from *T. asperellum* TA1 was done with the NucleoBond®HMW (DNA Macherey-Nagel™, Macherey-Nagel, Düren, Germany) kit according to the manufacturer’s protocol (Enzymatic lysis) and further purified via the isopropanol precipitation method described in [26], i.e., the same as described for the phenol–chloroform extraction after elution from the genomic tip. All samples were subjected to short read elimination via the Circulomics Short Read Eliminator XS kit (Circulomics, Part Number: SS-100-121-01). DNA purity was evaluated with NanoDrop One and concentration was measured with Qubit 4 resulting in 2.4 μg–6.7 μg HMW DNA per fungus. DNA length was determined on an Agilent TapeStation 4150 with Genomic DNA ScreenTapes. Library prep and barcoding was done according to the manufacturers protocol with the Native Barcoding Kit 24 V14 with an input of 400 ng per fungus, which was loaded on a PromethION Flow Cell (R10.4.1). Sequencing was done on a PromethION 24 with MinKNOW and run for 72 h with five other fungi, not part of this study, the samples were live basecalled with Super accurate basecalling Guppy (v7.1.4) [27] on a local machine, trimmed, and de-multiplexed in pod5 files for later basecalling with the Dorado [28].

### 2.2. Snakemake Workflow for High-Quality Assembly

**Figure 1:**
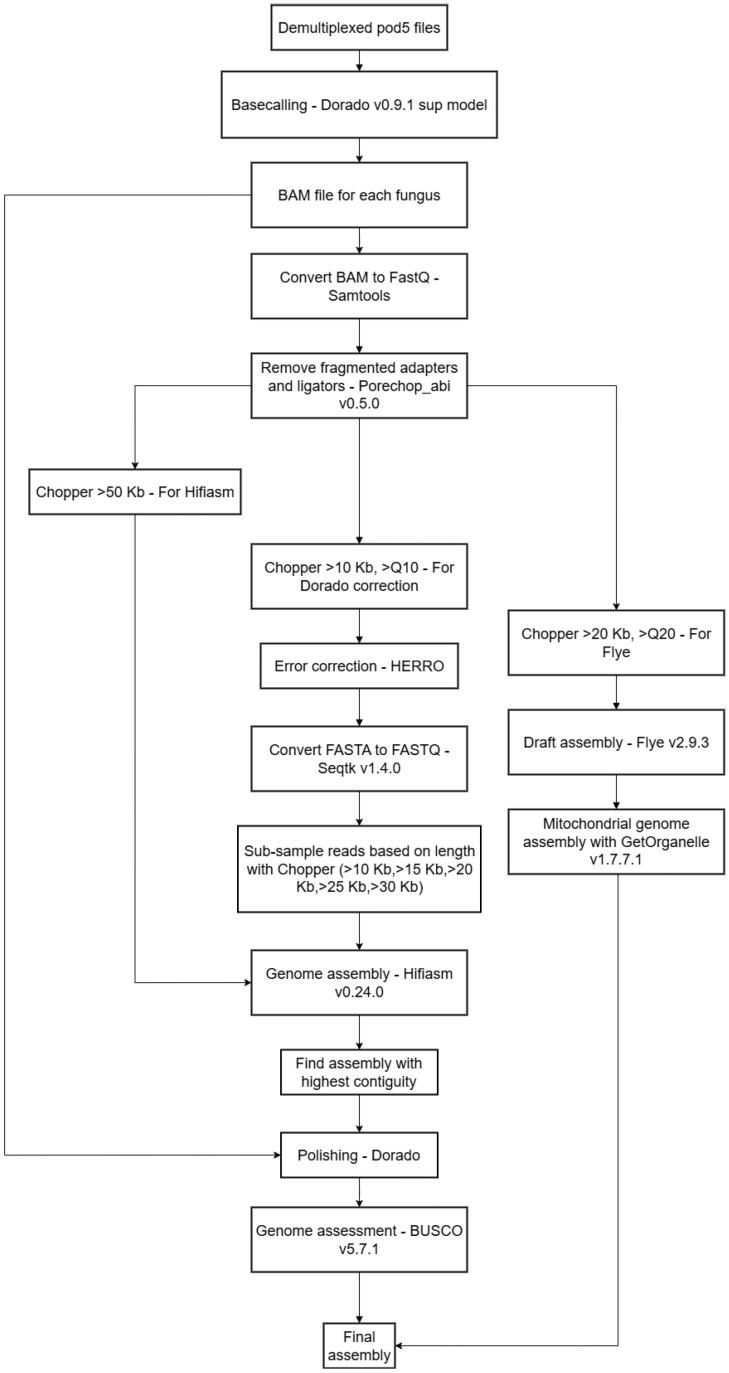
Overview of Snakemake workflow.

All computation was done on a high-performance computing (HPC) cluster running Slurm. The assembly pipeline was run in a custom snakemake (v7.18.2) [29] workflow. A general overview is visualized in Figure 1. Basecalling was performed with Dorado basecaller (v0.9.1) [28] using the sup model (v5.0.0) from the de-multiplexed pod5 files. SAMtools (v1.21) was used to convert Bam files to fastq files [30]. Porchop_abi (v0.5.0) [31] was used to remove fragmented adapters and ligators. Chopper (v0.9.0) [32] was used to filter reads based on length and quality; >Q10 and >10 Kb as the input for Read error correction, as per the recommendation described in [11]; >Q20 and >20 Kb as the input for Flye (v2.9.3) [33] based on preliminary experimentation that showed high contiguity for the assemblies, but also a robust assembly, as no assemblies failed at this filtering parameter; >Q10 and >50 Kb as the ultralong reads of one of the inputs for Hifiasm (v0.24.0) [34]. Flye (v2.9.3), was used to make draft assemblies for mitochondrial genome extraction for the assembly graph input of GetOrganelle (v1.7.7.1) [35]. Before Read error correction, Rasusa (v2.1.0) [36] was used to limit the reads to a maximum coverage of 125x for a genome of 40 Mb. HERRO [11] was run as implemented in the Dorado workflow for error correction of reads with Dorado correct (v0.9.1). Seqtk (v1.4.0) [37] was employed to convert fasta files from HERRO to fastq. Error-corrected reads were then filtered based on the lengths >10 Kb, >15 Kb, >20 Kb, >25 Kb, and >30 Kb with seqkit (v2.9.0) [38], and these 5 different read bins were then used, together with the ultralong reads, to assemble draft genomes, with Hifiasm (v0.24.0) [34] run in default mode as initial experimentation showed no need for changing parameters to produce T2T/near-T2T assemblies. Draft assemblies from Hifiasm (v0.24.0) were converted from fga files to fa files with a bash command. From these five draft assemblies obtained for each fungus, the draft assembly with the highest contiguity for each fungus was identified with a bash command and selected for polishing. The draft genome selected for polishing was then aligned with the bam file from the basecalling step with Dorado aligner (v0.9.1), sorted and indexed with SAMtools (v1.21); this was then used for polishing with Dorado polish (v0.9.1). The final assemblies were assessed with BUSCO (v5.7.1) [39] using the fungi_odb10 (01/08/24) dataset, run in genome mode. The full workflow and dependencies are available at https://github.com/TerpmikaelAAU/T2T_fungal_genome_pipeline (accessed on 6 August 2025). All other computation methods were run with either bash or python3.

### 2.3. Phylogenetic Determination with Universal Fungal Core Genes

Phylogeny was determined with the UFCG (v1.0.6) pipeline with default settings [40]. All reference genomes were downloaded from NCBI for their respective genus (24/03/2025) with NCBI Datasets (v16.2.0) [41]. For the *Neurospora* phylogeny, the four HQ (high-quality) assemblies of *N. sitophila* [42] were also included, as there were no *N. sitophila* genomes available in the NCBI database. A phylogenetic tree with Genealogical Sorting Index (GSI) values was visualized in iTOL (v7.2.1) [43]. For *T. asperellum* TA1, initial phylogeny was not sufficient to determine the specific species; therefore, the closest neighbors were used in a second run on the UFCG pipeline.

### 2.4. Whole-Genome Alignment

Whole-genome alignment was done with the closest related HQ genome with MUMmer (v3.23) [44] visualized with pyCirclize (v1.9.0) [45], a python implementation of Circos [46]. Mitochondrial genomes were aligned with NCBI blastn [47], with default parameters.

### 2.5. Gene Prediction and Functional Annotation

Ab initio gene prediction and subsequent functional annotations were conducted with the Funannotate (v1.8.17) pipeline [48]. Including antiSMASH 7.0 (v7.1) [49] and InterProScan (v5.73-104.0) [50,51]. Telomeres were identified via tidk (v0.2.63) [52] by searching with the experimental validated telomere sequences in Telobase (17/04/25) [53]. Functional genes and telomeres were visualized with pyCirclize (v1.9.0). Mitochondrial gene prediction was performed with MFannot [54] (8/8/2025), as Funannotate does not include mitochondrial gene prediction.

### 2.6. Validation of Workflow

To validate the Snakemake workflow, the basecalled reads of *Colletotrichum lini* 394-2 [12] was downloaded from the Sequence Read Archive. The workflow for these reads was started after the Porchop_abi step, no polishing was done, and the assembly quality was validated with the BUSCO (v5.3.2) glomerellales_odb10 (08/01/24) dataset run with default parameters in genome mode.

## 3. Results

### 3.1. Snakemake Workflow Output and Assembly Stats

The raw basecalled reads for the three different fungi were of high depth and quality, with the average Phred score being >Q20 for all the samples and an N50 of >10 Kb. After the first filtering step to prepare the reads for error correction, the coverage was halved for *N. intermedia* NRRL 2884 and *A. oryzae* CBS 466.91 and was lost for *T. asperellum* TA1 25%. After this, the samples displayed N50 > 20 Kb. Some reads were lost after error correction, and as the output of the Dorado error correction is in the fasta format, the quality scores are lost. Sub-sampling reads for the highest contiguity draft genome resulted in a much lower coverage for *N. intermedia* NRRL 2884 compared to *A. oryzae* CBS 466.91 and *T. asperellum* TA1. The ultra-long non-corrected reads had a coverage between 7.5 and 9.4 (Table 1). The percentage of reads > Q30 was 85% or more in all samples after basecalling; however, most of these were below 10 Kb and not included in further analysis. We observed that by not limiting the error correction step to reads below 50 Kb, higher contiguous draft assemblies were produced.

The BUSCO assessment of all genomes resulted in complete BUSCO scores > 98% for all samples and a genome size in congruence with what has been observed in the literature [55,56] (Table 2). All assemblies resolved into genomes with the same count of contigs as observed chromosomes in each fungus, without the mitochondrial genome. However, this was not the case for the *N. intermedia* NRRL 2884 assembly, as it had a mitochondrial contig with a size of 258 Kb, and no circular consensus of the mitochondrial genome could be assembled by Getorganelle in the Snakemake workflow. Manually rerunning Getorganelle on the output graph from Hifiasm did not produce a circular consensus either. In contrast, mitochondrial genomes were assembled automatically with Flye and Getorganelle for *T. asperellum* TA1 and *A. oryzae* CBS 466.91.

### 3.2. Phylogenetic Determination

**Figure 2:**
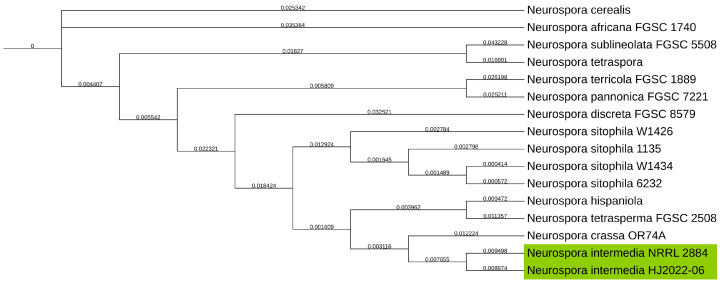
Phylogenetic tree of NCBI GenBank reference genomes of the *Neurospora* genus, with the *Neurospora intermedia* NRRL 2884 assembly produced in this study, clustering with *N. intermedia* HJ2022-06, highlighted in green, and not *N. sitophila* as it was first identified and acquired as from the strain collection. Genealogical Sorting Index (GSI) values shown on branches.

To determine/verify the taxonomic classification of the *N. intermedia* NRRL 2884 strain, it was aligned with the Universal Fungal Core Genes (UFCG) from UFCG. The produced tree showed that, instead of being grouped in the *sitophila* clade, it was grouped with *Neurospora crassa* and related most closely to *N. intermedia*, as shown in Figure 2.

**Figure 3:**
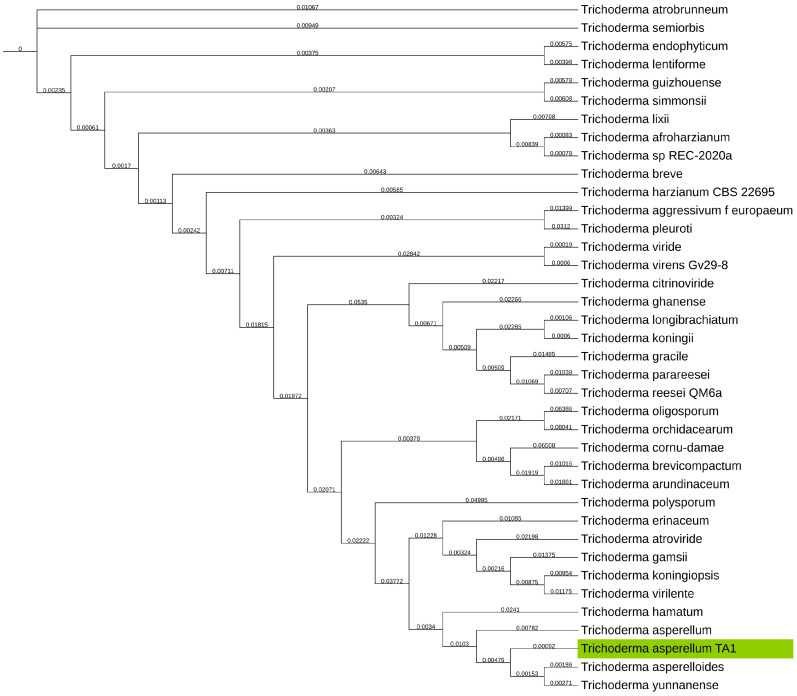
Phylogenetic tree of NCBI GenBank reference genomes of the *Trichoderma* genus, with the *T. asperellum* TA1 assembly from this study clustering close to *T. asperelloides*, *T. yunnanense*, and *T. asperellum*. *T. asperellum* TA1 assembly produced in this study highlighted in green. GSI values shown on branches.

With prior ITS classification performed on *T. asperellum* TA1, the taxonomical tree was also produced for verification/determination of the taxonomical classification. This was, however, not possible, as the *T. asperellum* TA1 assembly clustered close to *T. asperellum* and *T. asperelloides*, as shown in Figure 3, and therefore, there was no clear delineation of this fungus.

**Figure 4:**
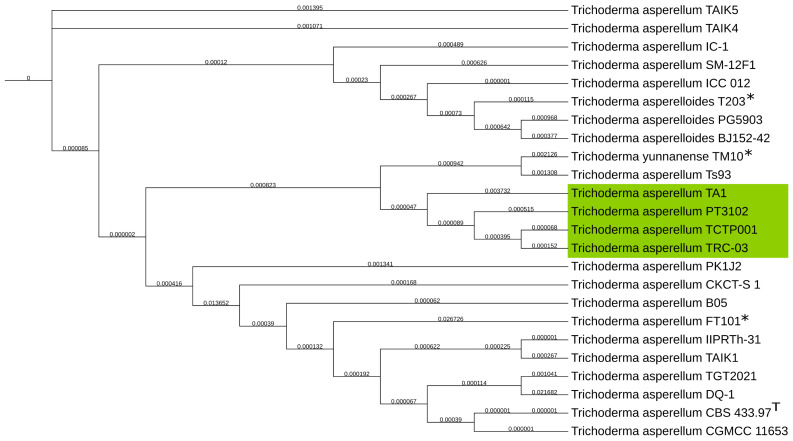
Phylogenetic tree of NCBI genomes of *T. asperelloides*, *T. yunnanense* and *T. asperellum*, for a higher resolution taxonomical classification. With the *T. asperellum* TA1 assembly produced in this study clustering closest to *T. asperellum* highlighted in green. GSI values shown on branches. NCBI GenBank reference genomes are marked with *. *T. asperellum* type strain is marked with T.

To achieve a higher resolution of taxonomical classification, the genomes available in the NCBI database of *T. asperellum*, *T. asperelloides*, and *T. yunnanense* were used for the alignment. Here, a clearer classification was observed of the *T. asperellum* TA1 assembly, being that of the *T. asperellum* species, as shown in Figure 4. Our phylogenetic analysis thereby supports the initial classification via ITS sequencing. Moreover, the *T. asperellum* TA1 is in a distinct clade of *T. asperellum* that is relatively far removed from the type strain *T. asperellum* CBS 433.97.

**Figure 5:**
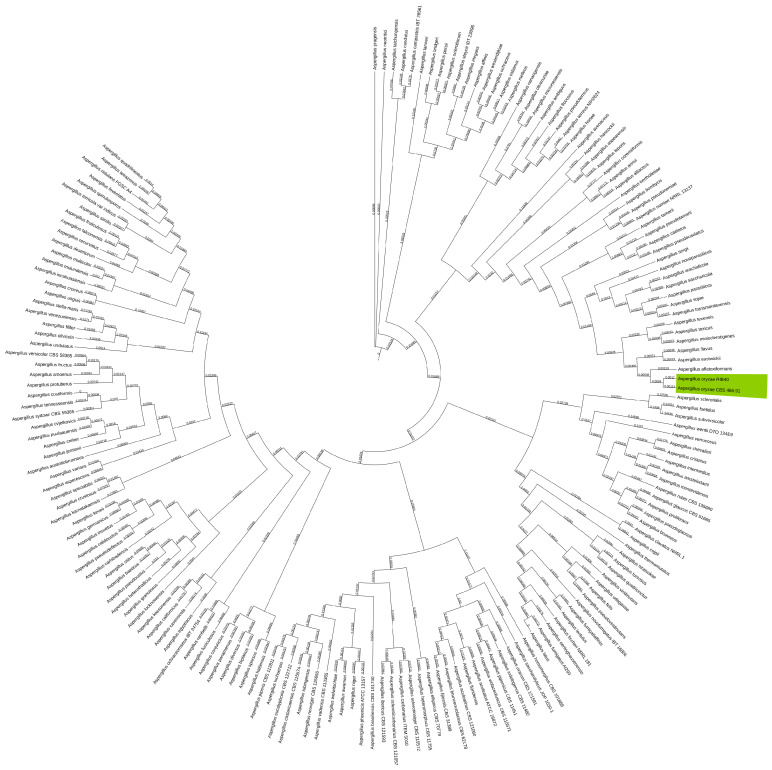
Phylogenetic tree of NCBI GenBank reference genomes of the *Aspergillus* genus showing the clustering of the *A. oryzae* CBS 466.91 genome assembled in this study clustering with *A. oryzae* RIB40 highlighted in green. GSI values shown on branches.

With *A. oryzae* being very closely related to *A. flavus*, which is known to produce aflatoxin [57], a clear taxonomic classification is imperative. In the alignment of all NCBI GenBank reference genomes of *Aspergillus*, the *A. oryzae* CBS 466.91 assembly produced in this study was neatly clustered with the reference strain of *A. oryzae* RIB40, as shown in Figure 5.

### 3.3. Whole-Genome Alignment with High Quality Closest Neighbor

**Figure 6:**
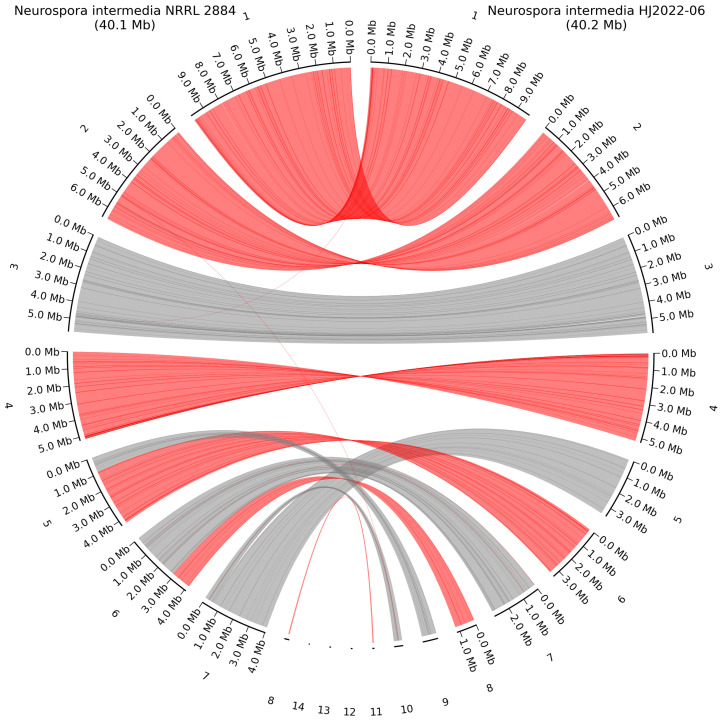
Whole-genome alignment of *N. intermedia* NRRL 2884 and the closest related HQ genome *N. intermedia* HJ2022-06 in the NCBI GenBank database. Grey lines show direct alignment; red lines show reverse complement alignment. Contig numbering scheme is based on length and ordered from largest to smallest contig.

Whole-genome alignment was made between the *N. intermedia* NRRL 2884 and NCBI GenBank reference genome *N. intermedia* HJ2022-06 to identify potential chromosomal rearrangements. No rearrangements were observed between these two species; however, the higher contiguity of our assembly was evident, as several of the smaller contigs of strain HJ2022-06 were mapped to different parts of whole chromosomes (Figure 6). The large 258 Kb mitochondrial genome assembled for *N. intermedia* NRRL 2884 was mapped to the smaller 87 Kb mitochondrial genome of scaffold 11 from *N. intermedia* HJ2022-06. Aligning these two mitochondria with blastn showed a query cover of 98% and an identity of 99.98%. By rerunning filtering with new parameters > Q25, >10 Kb and using the assembly graph from Flye for the mitochondrial genome assembly with Getorgannelle, a mitochondrial genome of 56 Kb was achieved. This was in much closer congruence with the HQ mitochondrial genome in *N. crassa* OR74 of 64.8 Kb. An alignment of these two with blastn had a query cover of 94% and an identity of 99.92%.

**Figure 7:**
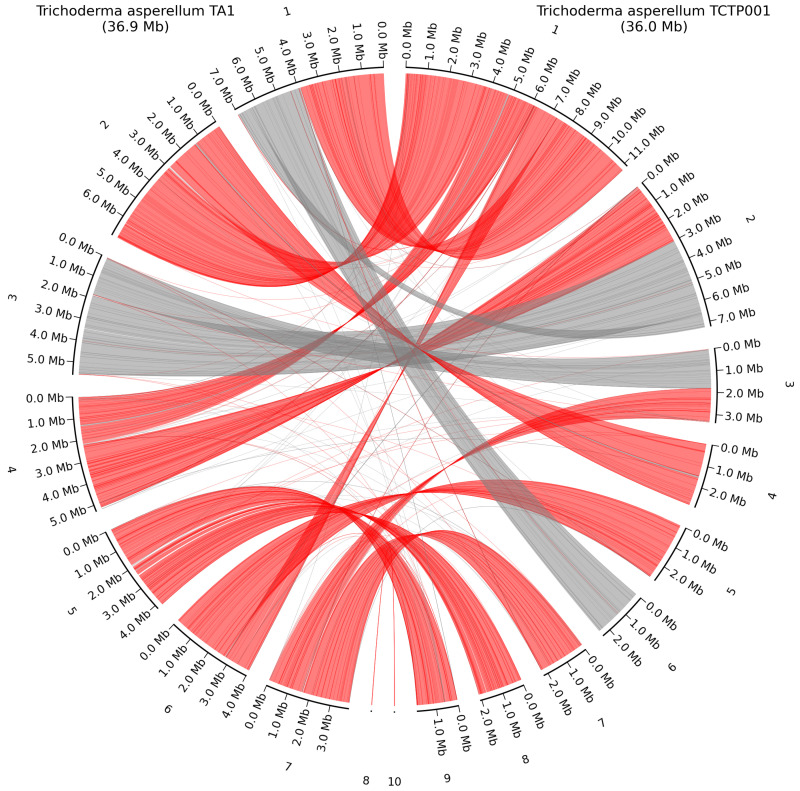
Whole-genome alignment of *T. asperellum* TA1 and the closest related HQ genome *T. asperellum* TCTP001. Grey lines show direct alignment; red lines show reverse complement alignment. Contig numbering scheme is based on length and ordered from largest to smallest contig.

Whole-genome alignment of *T. asperellum* TA1 and *T. asperellum* TCTP001 again show how some of the smaller contigs in *T. asperellum* TCTP001 are part of the fully assembled chromosomes in our assembly. However, there is a high number of chromosomal rearrangements either originating from the assembly method, or from chromosomal rearrangements events, regions represented by red lines indicate alignments in reverse orientation, reflecting potential inversions or structural rearrangements between the strains (Figure 7). The mitochondrial genomes shared high similarity as *T. asperellum* TA1 had a size of 29.7 Kb resembling the *T. asperellum* TCTP001 mitochondrial genome of 28.5 Kb, and alignment with blastn gave a query cover of 96% and a percent identity of 99.79%.

**Figure 8:**
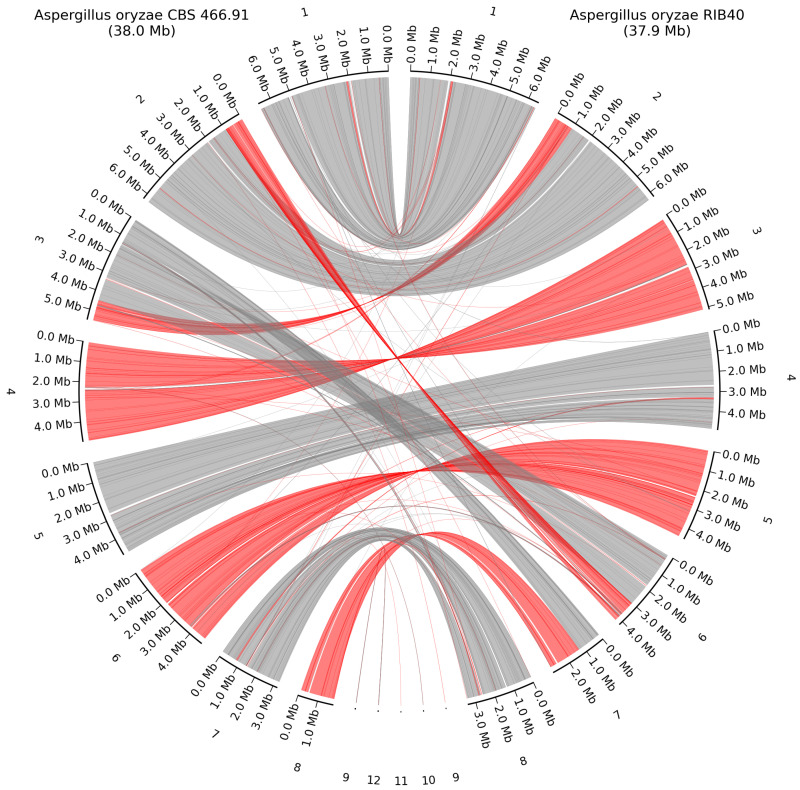
Whole-genome alignment of *A. oryzae* CBS 466.91 and the closest related HQ genome *A. oryzae* RIB40. Grey lines show direct alignment; red lines show reverse complement alignment. Contig numbering scheme is based on length and ordered from largest to smallest contig.

Some rearrangements were observed between *A. oryzae* CBS 466.91 and *A. oryzae* RIB40, with some of them being small parts of the genome and others being 1 Mb parts of the chromosomes, aligned to different chromosomes between the two strains, as seen on Figure 8. The three unplaced scaffolds from *A. oryzae* RIB40 each mapped to different chromosomes in *A. oryzae* CBS 466.91, 9 mapped to chromosome 2, 10 mapped to chromosome 4, and 11 mapped to chromosome 7.

The mitochondrial genome of *A. oryzae* CBS 466.91 and *A. oryzae* RIB40 were similar in size spanning 29.1 Kb and 29.2 Kb, respectively. Aligning the two mitochondrial genomes with blastn resulted in a percent identity 100% and a query coverage of 99.93%

### 3.4. Predicted Genes and Telomeres

Funannotate gene prediction and annotation identified 8790 genes in *N. intermedia* NRRL 2884, including 345 CAZymes, 8 PKSs, 5 NRPSs/NRPSs-like genes, 3 terpene synthases, and 4 RiPP clusters (Table 3). In comparison, *T. asperellum* TA1 had 9805 genes and *A. oryzae* CBS 466.91 had 13,042 genes, both showing a higher amount of BGCs (Table 3).

Mitochondrial genes were predicted with MFannot (Table 4). Comparing with mitochondrial genomes for their respective species showed close to the same genome size, GC content, tRNA, and rRNA [58,59,60].

**Figure 9:**
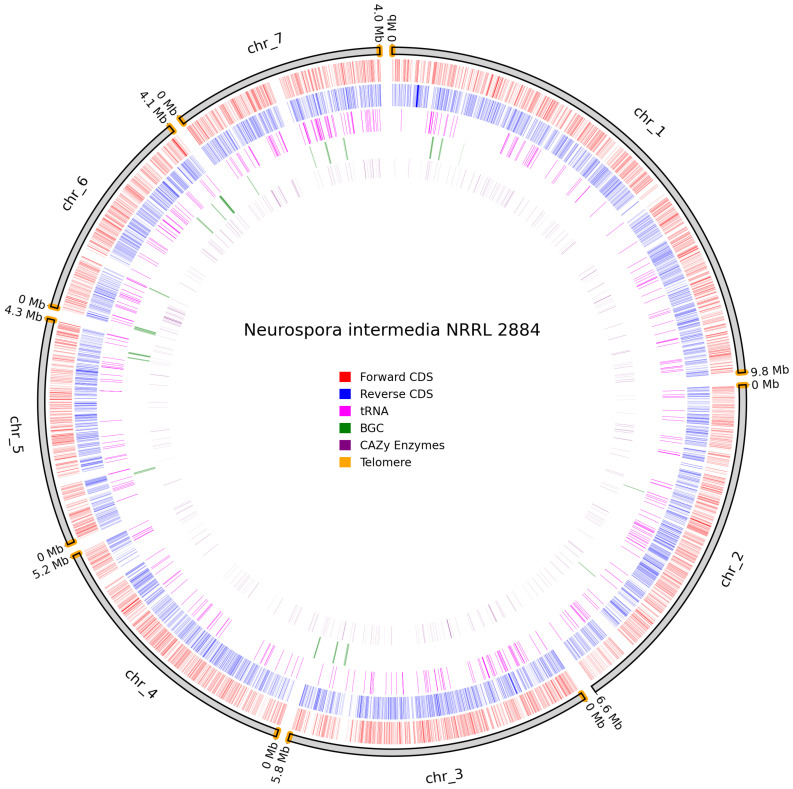
Overview of predicted annotation in *N. intermedia* NRRL 2884. Chromosome-naming scheme on the outer track, with chromosome size in Mb. Identified telomeres are colored in orange. Forward Coding sequence (CDS) in red, Reverse CDS in blue, tRNAs in pink, biosynthetic gene clusters (BGCs) in green, and carbohydrate-active enzyme (Cazyme) genes in purple.

*N. intermedia* NRRL 2884 assembled to a gapless near-T2T assembly, as it lacked one telomere on chromosome 2 but had identifiable telomeres on all other chromosomes, and a clear lack of CDS in chromosomes 1, 5, 6, and 7, which indicates that the centromeric regions are assembled correctly [61], as illustrated in Figure 9.

**Figure 10:**
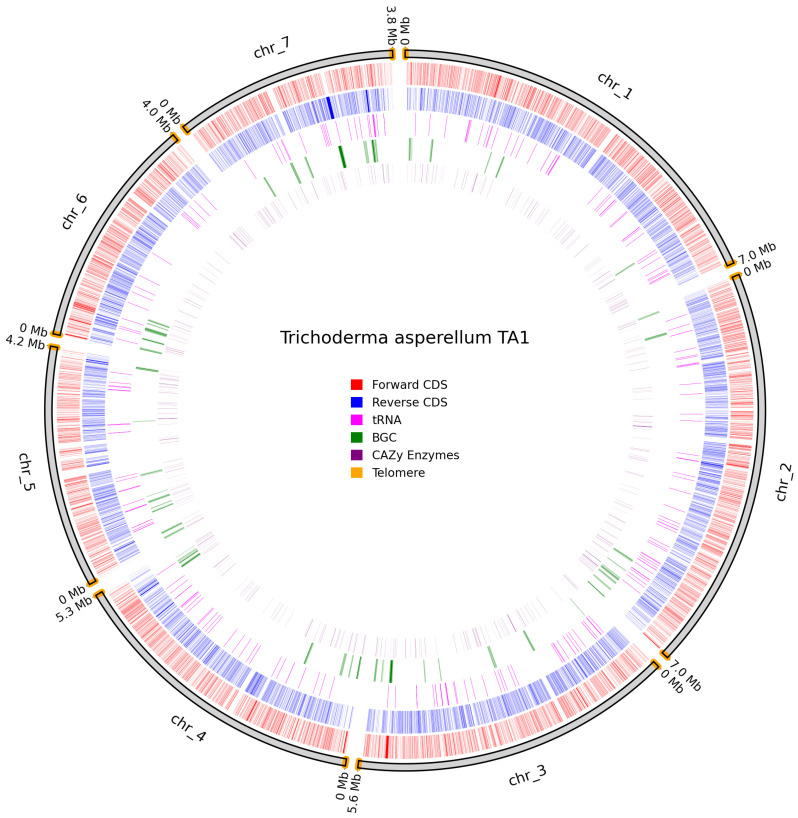
Overview of predicted annotation in *T. asperellum* TA1. Chromosome naming scheme on the outer track with chromosome size in Mb. Identified telomeres are coloured in orange. Forward CDS in red, Reverse CDS in blue, tRNAs in pink, BGCs in green, and Cazy genes in purple.

Telomeres were identified on all chromosomes of *T. asperellum* TA1; however, in this assembly, the centromeric regions are less clearly defined than in *N. intermedia* NRRL 2884 (Figure 10).

**Figure 11:**
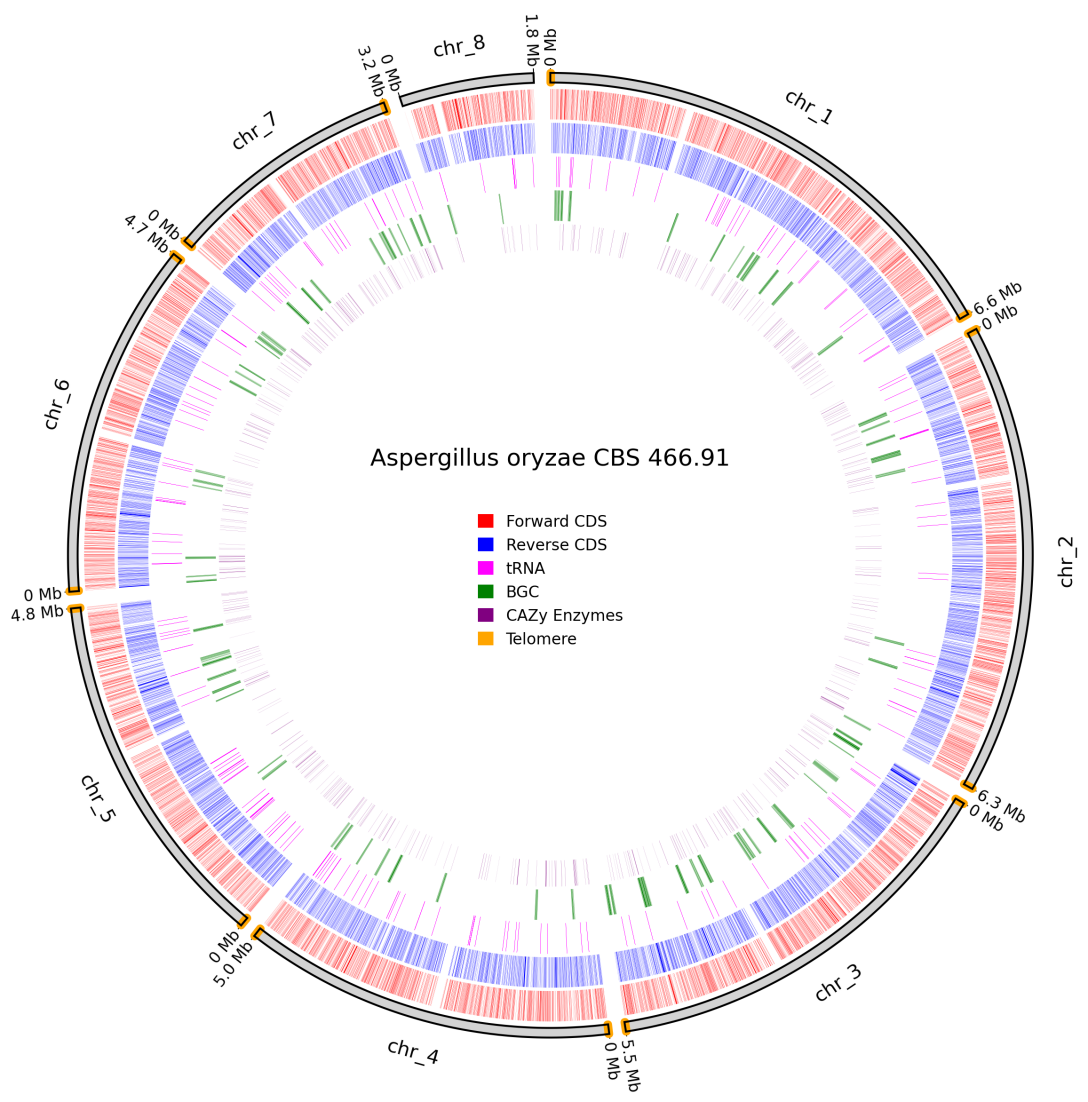
Overview of predicted annotation in *A. oryzae* CBS 466.91. Chromosome-naming scheme on the outer track with chromosome size in Mb. Identified telomeres are colored in orange. Forward CDS in red, Reverse CDS in blue, tRNAs in pink, BGCs in green, and Cazy genes in purple.

*A. oryzae* CBS 466.91 assembled to a gapless near-T2T assembly as it lacked telomeres on chromosome 8, but all other chromosomes contained telomeres on both ends (Figure 11). Despite the lack of telomeres, chromosome 8 is interpreted as a real chromosome, as it aligns with chromosome 7 in *A. oryzae* RIB40 and retains annotated functional genes.

### 3.5. Workflow Validation

The workflow presented in this study was tested with the reads of *Colletotrichum lini* 394-2. The two assemblies yielded identical BUSCO scores, with the only difference being a slightly smaller genome size in the assembly generated using the method described in this study (Table 5). In this case, the mitochondrial genome was produced during the initial Hifasm assembly rather than by GetOrganelle. These results further demonstrate that the approach presented here can generate fungal T2T assemblies solely from Nanopore simplex sequencing data, without the need for complementary sequencing from other platforms.

## 4. Discussion

The basecalling, filtering, read error correction, draft assembly, polishing, and mitochondrial genome recovery were performed in a near-fully automated manner by the newly developed Snakemake workflow. This approach yielded a near-T2T gapless genome for *Neurospora intermedia* NRRL 2884 and *Aspergillus oryzae* CBS 466.91, and a fully gapless T2T genome for *Trichoderma asperellum* TA1, all generated exclusively from Nanopore simplex sequencing data.

There is a pressing need to produce higher-quality fungal genomes, as 17,789 fungal genomes are currently available in the NCBI database, of which 91.6% are assembled only to the scaffold or contig level [62]. The ability to assemble fungal genomes to a gapless chromosome level, or to chromosome level, with a single, relatively inexpensive sequencing approach holds significant potential for increasing the overall quality of fungal genome assemblies in the future.

The workflow was validated by assembling the genome of *Colletotrichum lini* 394-2 [12], yielding a genome of comparable quality to that reported by the original authors. To our knowledge, this remains the only published study describing T2T fungal genome assemblies generated exclusively from Nanopore simplex data. In our implementation, the mitochondrial genome was assembled directly from the provided dataset, whereas Sigova et al. [12] incorporated the mitochondrial genome from a previous assembly because mitochondrial reads had been lost during the error-correction process, which was also observed in the present study for *T. asperellum* TA1 and *A. oryzae* CBS 466.91.

The mitochondrial genome of *N. intermedia* NRRL 2884 was initially assembled with corrected reads by Hifasm as a 258 Kb genome, many times larger than a typical *Neurospora* mitochondrial genome [58], and no contiguous mitochondrial genome was recovered in the initial GetOrganelle assembly. This initial 258 Kb mitochondrial sequence assembled for *N. intermedia* NRRL 2884 may contain NUMTs (nuclear mitochondrial DNA segments) [63,64], as most of the overrepresented mitochondrial DNA is lost in the HERRO Error correction step and would increase the likelihood of NUMTs being incorporated in the graph path. To address this anomaly, the filtering parameters for uncorrected reads in the Flye assembly were modified to a stricter filtering of Q25 instead of Q20, resulting in a less complicated assembly graph, as GetOrganelle is highly dependent on the quality of the assembly graph [35]. GetOrganelle could recover a mitochondrial genome that is much more consistent with that of a typical fungal mitochondrial genome. In contrast, the mitochondrial genomes of *T. asperellum* TA1 and *A. oryzae* CBS 466.91 were successfully assembled by GetOrganelle without such adjustments. For *C. lini* 394-2, mitochondrial reads were not lost during the error correction step and were correctly assembled by Hifasm. These findings highlight that mitochondrial genome assembly using this approach can be complex and may require further methodological refinement.

Accurate phylogenetic determination is often necessary to confirm or revise prior taxonomic classifications. While whole-genome alignments between species provide a more robust alternative to single-gene approaches such as UFCG [40] or BUSCO [39], such analyses impose substantial computational demands, particularly when handling large numbers of genomes [65]. Consequently, the use of multiple single-copy orthologs, as implemented in UFCG, remains the most practical strategy for phylogenetic analysis of newly assembled high-quality genomes. As observed in *Trichoderma* spp., greater resolution may be required for accurate species-level determination, likely due to the low synteny within the genus [66].

High-molecular-weight (HMW) DNA extraction is critical for next-generation sequencing. In this study, the phenol–chloroform extraction method successfully produced HMW DNA of suitable quality for *N. intermedia* NRRL 2884 and *A. oryzae* CBS 466.91, but not for *T. asperellum* TA1. For the latter, a commercially available kit employing enzymatic lysis followed by cetyltrimethylammonium bromide (CTAB) purification was used, yielding HMW DNA of sufficient quality for sequencing.

Using Snakemake, we provide tools for accurate assembly of complex fungal genomes with the aim of enabling future sequencing efforts to proceed more efficiently and at a faster pace.

## 5. Conclusions

In this study, we developed a reproducible Snakemake workflow capable of generating high-quality, gapless near-telomere-to-telomere fungal genome assemblies from Oxford Nanopore simplex data. The workflow streamlines the process of producing complete fungal genomes while minimizing manual intervention.This approach provides a standardized framework for fungal genomics research, improving both accessibility and reproducibility. Beyond facilitating high-resolution studies of fungal biology, pathogenicity, and evolution, the workflow also offers a scalable solution that can be adapted to a broad range of species and sequencing projects. Overall, our pipeline should lower the barrier to generating fungal T2T assemblies from Nanopore simplex data, supporting future efforts in comparative genomics, functional studies, and applications in medicine, agriculture, and biotechnology.

## Figures and Tables

**Table 1:** Read statistics for the snakemake workflow basecalling, filtering, and error correction results. Coverage was calculated based on the final genome assembly. Average quality was not calculated after error correction and the subsequent subsampling, as there is no intrinsic quality score after error correction.

Genus	Method	Coverage [X]	N50 [Kb]	Average Quality
*Neurospora*	Raw-Basecalled	194.56	11.375	20.31
*Neurospora*	Pre-Correction	104.67	21.338	20.58
*Neurospora*	Corrected	94.05	20.317	n/a
*Neurospora*	Contiguity	33.68	34.920	n/a
*Neurospora*	Ultra-long	7.53	59.306	20.14
*Neurospora*	Flye	42.53	30.951	23.34
*Trichoderma*	Raw-Basecalled	66.34	22.114	20.35
*Trichoderma*	Pre-Correction	49.06	29.752	20.56
*Trichoderma*	Corrected	44.78	27.294	n/a
*Trichoderma*	Contiguity	43.60	27.993	n/a
*Trichoderma*	Ultra-long	9.37	61.816	20.27
*Trichoderma*	Flye	26.45	37.005	23.45
*Aspergillus*	Raw-Basecalled	238.93	11.980	20.94
*Aspergillus*	Pre-Correction	136.76	19.340	21.13
*Aspergillus*	Corrected	118.29	18.200	n/a
*Aspergillus*	Contiguity	114.65	18.633	n/a
*Aspergillus*	Ultra-long	7.60	59.144	20.40
*Aspergillus*	Flye	51.27	29.417	23.74

**Table 2:** BUSCO results. C (complete BUSCO), S (Single copy BUSCO), D (Duplicated BUSCO), F (Fragmented BUSCO), M (Missing BUSCO), n (number of genes in BUSCO dataset), DB (BUSCO dataset).

Genus	Contigs	Size [Mb]	C [%]	S [%]	D [%]	F [%]	M [%]	n	DB
*Neurospora*	7(+1) ^1^	40	99.4	99.1	0.3	0.3	0.3	758	fungi_odb10
*Trichoderma*	7	37	98.8	98.5	0.3	0.3	0.9	758	fungi_odb10
*Aspergillus*	8	38	98.7	98.3	0.4	0.5	0.8	758	fungi_odb10

^1^ The extra contig was a wrongly assembled mitochondrial genome

**Table 3:** Genes, core enzymes, and BGCs predicted for the fungal genomes.

Strain	Genes	CAZymes	PKSs	NRPSs/NRPSs-like	Terpene Synthases	RiPPs	BGCs
*N. intermedia* NRRL 2884	8790	345	8	5	3	4	21
*T. asperellum* TA1	9805	401	17	18	10	3	51
*A. oryzae* CBS 466.91	13,042	579	28	32	12	6	78

**Table 4:** Mitochondrial genomes overall statistics.

Strain	Size [Kb]	GC Content [%]	Protein CDS	tRNA	rRNA
*N. intermedia* NRRL 2884	56.3	36	31	27	1
*T. asperellum* TA1	29.7	28	18	27	1
*A. oryzae* CBS 466.91	29.1	26	21	28	1

**Table 5:** BUSCO results. C (complete BUSCO), S (Single copy BUSCO), D (Duplicated BUSCO), F (Fragmented BUSCO), M (Missing BUSCO), and DB (BUSCO dataset).

Sample	Contigs	Size [Mb]	C [%]	S [%]	D [%]	F [%]	M [%]	DB
*C. lini* 394-2 ^1^	13	53.56	96.8	96.6	0.2	0.6	2.6	glomerellales_odb10
*C. lini* 394-2	13	53.69	96.8	96.6	0.2	0.6	2.6	glomerellales_odb10

^1^ Assembled in this study.

## Data Availability

The sequencing data and genomes produced in this study can be found in the NCBI database under the BioProject accession number: PRJNA1302126. The snakemake workflow and other code used in this study can be found at https://github.com/TerpmikaelAAU/T2T_fungal_genome_pipeline (accessed on 6 August 2025).

## Abbreviations

The following abbreviations are used in this manuscript:

BGCs	Biosynthetic gene clusters
BUSCO	Benchmarking Universal Single-Copy Orthologs
CAZymes	Carbohydrate-Active Enzymes
CTAB	Cetyltrimethylammonium bromide
GSI	Genealogical Sorting Index
CDS	Coding sequence
HERRO	Haplotype-aware error correction
HMW	High molecular weight DNA
HPC	High-performance computing
HQ	High quality
NRPSs	Nonribosomal peptide synthetases
NUMTs	Nuclear mitochondrial DNA segments
PKSs	Polyketide synthases
RiPPs	Post-translationally modified peptides
T2T	Telomere to telomere
Tidk	Telomere identification toolkit
UFCG	Universal Fungal Core Genes
YES	Yeast extract sucrose
YPG	Yeast extract Peptone Glucose
